# Effects of Multi-Session Repetitive Transcranial Magnetic Stimulation on Motor Control and Spontaneous Brain Activity in Multiple System Atrophy: A Pilot Study

**DOI:** 10.3389/fnbeh.2018.00090

**Published:** 2018-05-09

**Authors:** Zhu Liu, Huizi Ma, Victoria Poole, Xuemei Wang, Zhan Wang, Yaqin Yang, Lanxi Meng, Brad Manor, Junhong Zhou, Tao Feng

**Affiliations:** ^1^Department of Neurodegenerative Disease, Centre of Neurology, Beijing Tiantan Hospital, Capital Medical University, Beijing, China; ^2^Harvard Medical School, Harvard University, Roslindale, MA, United States

**Keywords:** repetitive transcranial magnetic stimulation, multiple system atrophy, motor control, multiscale entropy, resting-state fMRI

## Abstract

**Background:** Impaired motor control is one of the most common symptoms of multiple system atrophy (MSA). It arises from dysfunction of the cerebellum and its connected neural networks, including the primary motor cortex (M1), and is associated with altered spontaneous (i.e., resting-state) brain network activity. Non-invasive repetitive transcranial magnetic stimulation (rTMS) selectively facilitates the excitability of supraspinal networks. Repeated rTMS sessions have been shown to induce long-term changes to both resting-state brain dynamics and behavior in several neurodegenerative diseases. Here, we hypothesized that a multi-session rTMS intervention would improve motor control in patients with MSA, and that such improvements would correlate with changes in resting-state brain activity.

**Methods:** Nine participants with MSA received daily sessions of 5 Hz rTMS for 5 days. rTMS targeted both the cerebellum and the bilateral M1. Before and within 3 days after the intervention, motor control was assessed by the motor item of the Unified Multiple System Atrophy Rating Scale (UMSARS). Resting-state brain activity was recorded by blood-oxygen-level dependency (BOLD) functional magnetic resonance imaging. The “complexity” of resting-state brain activity fluctuations was quantified within seven well-known functional cortical networks using multiscale entropy, a technique that estimates the degree of irregularity of the BOLD time-series across multiple scales of time.

**Results:** The rTMS intervention was well-attended and was not associated with any adverse events. Average motor scores were lower (i.e., better performance) following the rTMS intervention as compared to baseline (*t*_8_ = 2.3, *p* = 0.003). Seven of nine participants exhibited such pre-to-post intervention improvements. A trend toward an increase in resting-state complexity was observed within the motor network (*t*_8_ = 1.86, *p* = 0.07). Participants who exhibited greater increases in motor network resting-state complexity demonstrated greater improvement in motor control (*r*^2^= 0.72, *p* = 0.004).

**Conclusion:** This pilot study demonstrated that a five-session rTMS intervention targeting the cerebellum and bilateral M1 is feasible and safe for those with MSA. More definitive, well-controlled trials are warranted to confirm our preliminary results that rTMS may alleviate the severity of motor dysfunction and modulate the multiscale dynamics of motor network brain activity.

## Introduction

Multiple system atrophy (MSA) is a fatal neurodegenerative disorder marked by progressive parkinsonian symptoms and both cerebellar and autonomic dysfunction. Up to 87% of patients with MSA exhibit motor disturbances, which in turn increase morbidity and reduce quality of life (Fanciulli and Wenning, 2015). Current pharmacological therapies targeting rigidity and bradykinesia, which most commonly entail anti-parkinsonism drugs (e.g., Levodopa) (Kollensperger et al., 2010), are largely ineffective (Krismer and Wenning, 2017). Only 31% of patients with MSA benefit from Levodopa, and such improvements tend to be short-lasting (Gilman et al., 2008; Maass et al., 2016; Rohrer et al., 2017). Moreover, these medications often cause side effects including hypotension, cognitive impairment, and hypersomnia in this population. There is thus an urgent need to develop new *non-pharmacological* therapeutic strategies to enhance motor function in those suffering from MSA.

Motor control impairments in MSA are believed to arise from dysfunction of the cerebellum and its connected neural networks (Payoux et al., 2010; Lu et al., 2013). MSA leads to atrophy in cerebellar regions, as well as altered plasticity and decreased neuronal excitability (e.g., prolonged central motor conduction time and cortical silent period) within the motor cortex (M1) (Fanciulli and Wenning, 2015). Burciu et al. (2016) observed in a longitudinal study of patients with MSA that the responsiveness [i.e., activation measured by functional magnetic resonance imaging (fMRI) signal] of the M1 and cerebellum to a motor task diminished over a 1-year period. This observation suggests that therapeutic strategies designed to modulate activity in the cerebellum and M1 may be particularly well-suited to slow the progression of functional decline, or even enhance it, in this population.

Non-invasive repetitive transcranial magnetic stimulation (rTMS) is a safe technique that selectively modulates the excitability of neuronal populations and their connected neural networks via current induced by an electro-magnetic field. Yildiz et al. (2017), for example, observed that rTMS targeting the cerebellum reduced short-latency afferent inhibition of M1 and enhanced performance of a cognitive reaction time task in those with MSA-cerebellar subtype (MSA-C). The multi-session rTMS intervention protocol has shown great promise to induce longer-term effects on the behavioral performance. Chang et al. (2010) observed that 10 sessions of daily rTMS induced improvement in motor function of subacute stroke patients and such improvement lasted for 3 months. Still, the effects of a multi-session rTMS intervention on motor control performance are largely unknown.

The functional regions of the brain are continuously interacting with each other over multiple temporal scales, even during “resting-state” (Khambhati et al., 2015). As such, the fluctuations of spontaneous brain activity, which can be recorded with blood-oxygen-level dependency (BOLD) fMRI, are “complex.” Here, complexity refers to the presence of information-rich fractal-like patterns within the time-series. The degree of such resting-state complexity within large-scale functional networks has been associated with functional performance. For example, Yang et al. (2013) reported that older adults who exhibited greater MSE-derived resting-state complexity within default mode network had better cognitive function.

In this study, we examined the effects of a five-session, daily rTMS intervention targeting both the bilateral cerebellum and M1 on motor control and the resting-state complexity of brain activity in a small sample of patients with MSA. We hypothesized that as compared to baseline, participants would demonstrate improved motor control, along with increased resting-state complexity, following the rTMS intervention. We further hypothesized that observed improvements in motor control would correlate with observed increases in resting-state complexity.

## Materials and Methods

### Participants

Nine participants with *de novo* MSA [four men; age (mean ± standard deviation): 58.0 ± 7.0 years, ranging from 50 to 66] were recruited from the Department of Neurodegenerative Disease, Center of Neurology, Beijing Tiantan Hospital, Capital Medical University (Beijing, China). The characteristics of each participant are summarized in **Table 1**. MSA was diagnosed according to Gilman et al. (2008) by two experienced clinicians. Three participants were diagnosed as MSA-parkinsonian subtype (MSA-P) and the other six presented with MSA-C. Exclusion criteria included contraindications to rTMS (i.e., significant medical or psychiatric illnesses, pregnancy, mental diseases, brain trauma, personal or family history of seizures or epilepsy, metallic or electrical bio-implants), contraindications to magnetic resonance imaging (MRI) (i.e., personal or family history of seizures or epilepsy, BMI > 40, metallic or electrical bio-implants, claustrophobia), and cognitive impairment as defined by a Mini-Mental State Examination (MMSE) score ≤ 24. None of these participants had responded well to previous Levodopa treatment.

**Table 1:** Participant demographics and clinical characteristics.

ID	MSA subtype	Age (years)	Sex	MSA duration (years)	IALT	Baseline motor score of UMSARS
1	P	53	M	4	0	19
2	P	57	F	3	22.58%	22
3	P	66	F	3	19.60%	23
4	C	51	F	2	0	19
5	C	50	F	1.5	0	26
6	C	66	M	2	0	12
7	C	66	M	2	0	18
8	C	62	F	2	0	22
9	C	51	M	2	0	17


*P, MSA-P; C, MSA-C; M, male; F, female; IALT, improvement rate of acute stepwise levodopa challenge test; UMSARS, Unified Multiple System Atrophy Rating Scale.*

### Ethics Statement

This study was approved by institutional review board of Beijing Tiantan Hospital, Capital Medical University and conducted according to the principles of the Declaration of Helsinki. The registration number of this study in Beijing Tiantan Hospital was KYSB2017-169-01. All participants provided written informed consent prior to screening and study participation.

### Study Protocol

All participants completed one daily session of rTMS targeting both the cerebellum and bilateral M1 over five consecutive days. Motor control and resting-state brain activity were assessed at baseline and within 3 days of the last rTMS session. All participants stayed within the Tiantan Hospital Clinical Center throughout the entire study. Pre- and post-intervention assessments were conducted at approximately the same time of day and a clinician confirmed the absence of any acute medical condition that may have interfered with functional performance. Dopaminergic treatments, if used, were withheld throughout the course of the study.

### Repetitive Transcranial Magnetic Stimulation (rTMS)

Participants received rTMS while seated in a chair with electromyography electrodes over the abductor pollicis brevis (APB) muscle to record motor evoked potentials (MEPs). A MagPro Compact stimulator (Dantec Medical, Copenhagen, Denmark) was connected to a 50-mm round coil (MCF-B65). The left and right targeting sites of M1 were determined as the location on the participant’s scalp where TMS evoked maximal MEPs in the contralateral APB. The targeting sites of the cerebellum were 3 cm lateral to the left of the inion and 3 cm lateral to the right of the inion. The resting motor threshold (RMT) was measured with the left M1 and then the delivered intensity was set to at 100% of the RMT. The rTMS course consisted of daily sessions of 500 pulses for each M1 target and 500 pulses for each cerebellum target (i.e., 2000 pulses and 50 trains at 5 Hz for 5 days). In each session, rTMS was delivered over the left and right M1 and cerebellum sequentially.

### Motor Control Performance

Motor control was assessed using the Motor Examination Scale within the Unified Multiple System Atrophy Rating Scale (UMSARS) (Wenning et al., 2004). This scale consists of 14 tests that measure multiple aspects of motor control (e.g., finger tapping, facial expression, posture, gait, etc.). Participant performance in each test was scored by an experienced clinician on a five-point scale ranging from 0 to 4, where lower scores reflected better performance. Scores on the 14 tests were summed and used for analysis.

### Magnetic Resonance Imaging

A Siemens Trio 3-Tesla scanner (Siemens, Erlangen, Germany) was used to acquire all MRI data. High-resolution brain structural images were acquired using T1-weighted, sagittal 3D magnetization-prepared rapid-gradient echo (MPRAGE) sequences with the following parameters: repetition time (TR)/echo time (TE)/inversion time = 2000 ms/2.19 ms/900 ms, flip angle (FA) = 9°, field of view (FOV) = 224 mm × 256 mm, in-plane resolution = 224 × 256, slice thickness = 1 mm, and 176 sagittal slices. Functional images were axially collected using an echo-planar imaging (EPI) sequence with the following settings: TR/TE = 2000 ms/40 ms, FA = 90°, FOV = 256 mm × 256 mm, resolution = 64 × 64, axial slices = 28, thickness/gap = 4 mm/1 mm, bandwidth = 2230 Hz/pixel.

### Data Analysis

#### Resting-State fMRI

Resting-state functional data were pre-processed using AFNI. The following steps were performed: volume registration, alignment to the T1 anatomy, warp into Talairach space, 8-mm kernel smoothing, and scaling to a percentage of the mean. A band-pass filter was used to remove fluctuations below 0.01 and above 0.08 Hz. Filtered data were enter into a general linear model to remove the effects of 6 degrees of motion, nuisance CSF, white matter, and global signal. The residual time series in each voxel from this deconvolve was then used to calculate multiscale entropy.

#### Multiscale Entropy (MSE)

**FIGURE 1:**
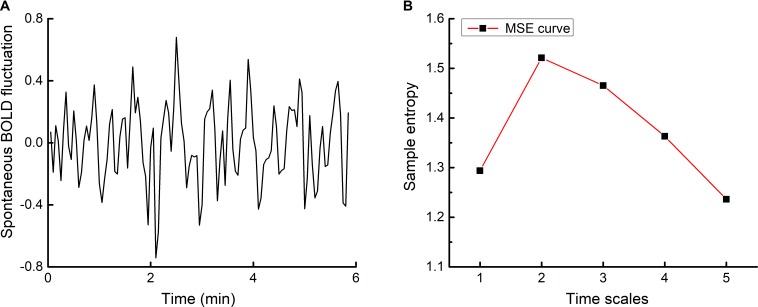
An example time-series of resting-state BOLD fluctuations within one brain voxel **(A)** and its corresponding multiscale entropy (MSE) curve **(B)**. The complexity of voxel-level BOLD fluctuations was determined by computing sample entropy across multiple time scales and averaging across said scales. Network-level resting-state complexity was then determined by parceling the brain into seven large-scale networks according to Yeo et al. (2011) and averaging the complexity values associated with all voxels within each network.

Multiscale entropy (MSE) (Costa et al., 2002) was used to quantify the complexity of the spontaneous BOLD time series in each brain voxel (**Figure 1A**). MSE is a widely-used technique for quantifying the degree of re-occurrence of repetitive behavior (or patterns) across multiple time scales in a bio-physiological time-series, such that series with less pattern re-occurrence are more complex. Here, complexity was quantified using time scales one through five, as follows. The BOLD time series of each voxel was “coarse-grained” on these five scales by averaging point values using non-overlapping windows of length equaling to the scale factor τ (i.e., τ = 1 to 5). For example, the time series used for scale one was the original (filtered) time-series. The time-series at scale three was constructed by averaging every three points in the original time-series. The sample entropy of each coarse-grained time series was then calculated. Sample entropy is defined by the negative natural logarithm of the conditional probability that a time-series, having repeated itself within a tolerance r for m points (defined pattern length), will also repeat itself for m + 1 points without self-matches. Here, we chose *m* = 1 and *r* = 0.35, based upon previous studies that used MSE to analyze BOLD time-series (Yang et al., 2013, 2014, 2015).

The length of each coarse-grained time series was equal to the length of the raw time series divided by the scale factor. The number of data points in the coarse-grained time series at the maximum scale thus equaled to 23 (i.e., 117 divided by 5), greater than the 10^m^ to 20^m^ points (i.e., 10 to 20 as *m* = 1) required for reliable estimation of sample entropy (Costa et al., 2002, 2005). The complexity index of each individual voxel BOLD time-series was then computed by averaging sample entropy across the five time scales (Yang et al., 2014) (see **Figure 1B**). Network-level resting-state complexity was calculated by parceling the brain into seven large functional networks (i.e., visual, motor, dorsal attention, ventral attention, limbic, executive, and default mode networks) according to Yeo et al. (2011). The parcellation was Talairach-normalized, resampled to 3 × 3 × 3 voxels, and separated into individual networks. Resting-state complexity of each network was defined as the average complexity of all voxels within each network. As such, greater values reflected greater resting-state complexity of a given network.

### Statistical Analysis

Analyses were performed with JMP Pro 12 software (SAS Institute, Cary, NC, United States). Primary outcomes were the UMSARS motor score and the resting-state complexity of the seven functional networks. Variable normality was examined with the Shapiro–Wilk *W*-test and homogeneity of variance was determined with the Levene test. Paired-*t* tests were used to analyze the effects of tTMS on each outcome.

Linear regression analyses were used to determine the relationship between rTMS-induced percent changes in motor control and the resting-state complexity of each network at baseline, as well as the percent change in complexity from pre-to-post intervention. Significance level was set to *p* < 0.05 for all analyses.

## Results

All participants completed the study and intervention compliance was 100%. Stimulation was well-tolerated by all subjects and was not associated with any self-reported adverse event.

### The Effects of rTMS Intervention on Motor Control

**FIGURE 2:**
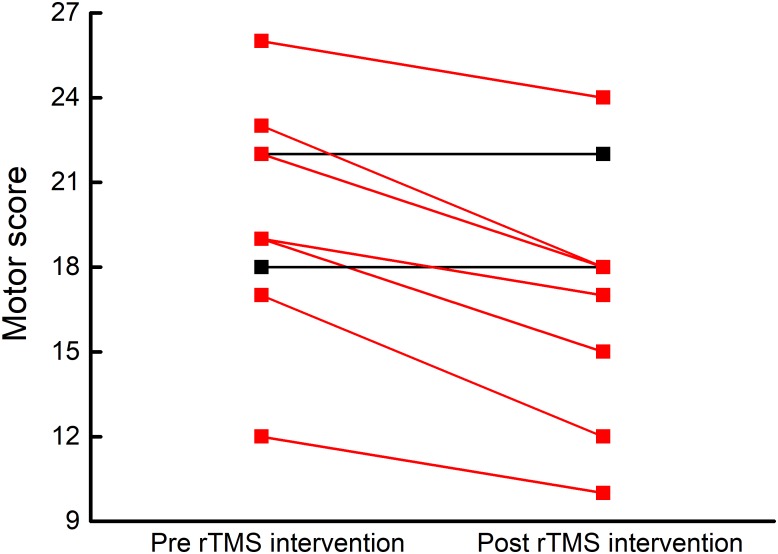
Participant-level motor control before and after rTMS intervention. Motor control was assessed by the Motor Examination Scale within the Unified Multiple System Atrophy Rating Scale (UMSARS), where lower scores reflect better motor control. After five, once-daily sessions of rTMS targeting the cerebellum and bilateral M1, seven of nine participants exhibited lower motor scores (red lines).

Motor scores at baseline and after intervention was normally distributed and exhibited homogeneity of variance. After the rTMS intervention, seven participants exhibited improved motor control (i.e., lower motor scores) as compared to baseline (**Figure 2**). At the group-level, the motor item score decreased from 19.8 ± 4.1 at baseline to 17.1 ± 4.4 after the intervention (averaged decrease: 2.7 ± 1.9) (*t*_8_= 2.3, *p* = 0.003).

### The Effect of rTMS Intervention on Resting-State Complexity

**FIGURE 3:**
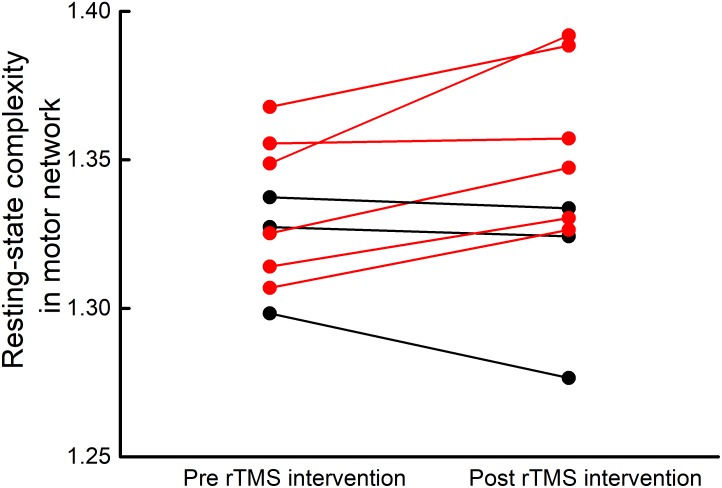
Participant-level motor network resting-state complexity before and after rTMS intervention. After five, once-daily sessions of rTMS targeting the cerebellum and bilateral M1, six of nine participants exhibited increased resting-state complexity within the motor network (red lines).

Resting-state complexity values of the seven brain functional networks at baseline and after intervention were normally distributed and exhibited homogeneity of variance. After the rTMS intervention, six participants demonstrated an increase in resting-stated complexity within the motor cortex (**Figure 3**). A trend toward increased pre-to-post intervention motor network resting-state complexity was observed at the group level (*t*_8_= 1.86, *p* = 0.07). No changes were observed in the resting-state complexity of the other six networks (*t*_8_< 1.7, *p* > 0.13).

### Relationship Between Motor Function and Resting-State Complexity

**FIGURE 4:**
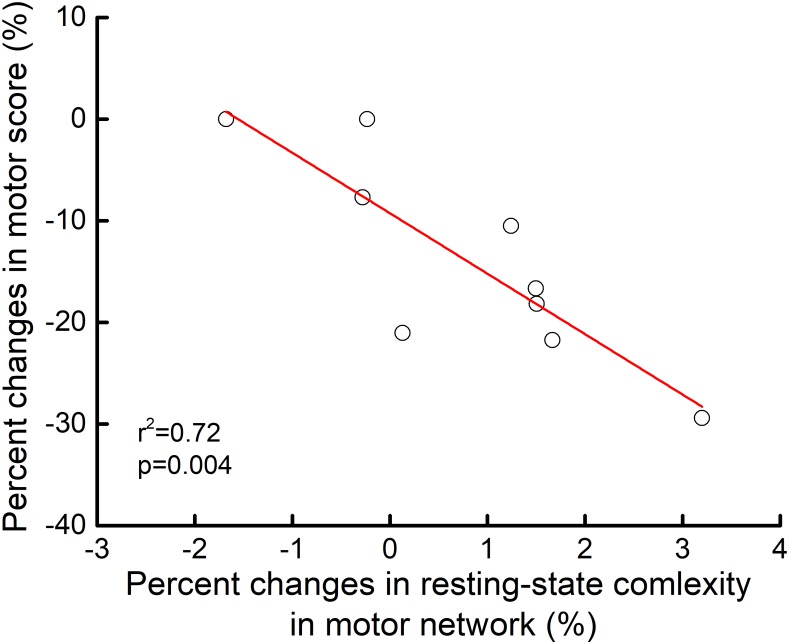
Relationship between pre-to-post rTMS intervention changes in motor control and motor network resting-state complexity. From pre-to-post intervention, participants who exhibited greater percent decrease in motor item score (i.e., better performance) demonstrated greater percent increase in resting-state complexity within the motor network (*r*^2^= 0.72, *p* = 0.04). No other relationships between motor control and resting-state complexity reached significance.

At baseline, motor scores did not correlate with the resting-state complexity of brain networks. However, linear regression analysis revealed that the percent change in motor score from pre-to-post intervention correlated with the pre-to-post percent change in motor network resting-state complexity (*r*^2^= 0.72, *p* = 0.004). Those who exhibited greater increases in motor control also experienced greater increases in resting-state complexity within the motor network (**Figure 4**).

## Discussion

Dysfunction of motor control is a severe pathological symptom of MSA and there are currently no effective strategies for alleviating such burden. This pilot study provided first-of-its-kind preliminary evidence that a five-session rTMS intervention targeting both cerebellum and bilateral M1 is feasible and may improve motor control in patients with MSA. Moreover, such improvements appear to be linked to an increase in the physiologic complexity of resting-state brain activity dynamics, specifically within the motor network. Together, these initial observations warrant more definitive, well-controlled studies to establish the effectiveness of rTMS on motor control and underlying brain function in this vulnerable population.

The cerebellum and its connected neural network, including the bilateral M1, are closely involved in motor control. In the cerebellum-M1 circuit, the excitability (i.e., likelihood of neuronal firing) of M1 is modulated by the dentate nucleus and Purkinje cells within the cerebellum (Grimaldi et al., 2014). MSA impairs regulation of both the dentate nucleus and Purkinje cells, resulting in diminished excitability of M1 and ultimately, motor control dysfunction. High-frequency rTMS stimulation facilitates neuronal excitability within targeted regions (Rossi et al., 2009; Lefaucheur et al., 2014) and such facilitation can induce functional improvements. Recent meta-analyses and systematic reviews indicate that, for example, high frequency rTMS targeting M1 induces mild-to-moderate motor improvements (Chou et al., 2015; Wagle Shukla et al., 2016) in Parkinson disease (PD). Similarly, high frequency rTMS targeting the cerebellum may reduce cerebellar inhibition by modifying the activity of the dentate nucleus and Purkinje cells in the cerebellum (Cury et al., 2015), of which the underlying mechanism is worthwhile to be explored in future studies.

Recent non-invasive brain stimulation studies have demonstrated that “multi-focal” interventions (i.e., stimulating multiple regions within one session) may be particularly beneficial to motor control. Spagnolo et al. (2014) reported that a 12-session rTMS intervention targeting both the M1 and prefrontal regions induced significant improvement of motor function in those with Parkinson’s disease, as evidenced by an 11 point average reduction in UPDRS scores. In the current study, rTMS targeted both the cerebellum and bilateral M1. This novel protocol may thus both directly increase the excitability of the M1 and reduce cerebellar inhibition of the M1. As the two regions are structurally and functionally connected, studies are needed to determine if targeting both regions have greater effects as compared to targeting either region separately.

Numerous studies have demonstrated that for a given physiological system, fluctuations within its spontaneous behavior are not random, but instead contain “meaningful” patterns over multiple scales of time (Lipsitz and Goldberger, 1992; Pikkujämsä et al., 1999; Manor and Lipsitz, 2013). The degree of complexity associated with these fluctuations reflects their “richness,” or information content, and has been linked to system functionality (Manor et al., 2010; Zhou et al., 2017). Within the current small cohort, the rTMS intervention was associated with a trend toward increased resting-state complexity, specifically within the motor network. Moreover, those who exhibited greater increases in complexity also tended to make larger improvements in motor control. While appropriately-powered sham-controlled studies are needed to confirm these preliminary results, these observations suggest that observed motor control improvements were likely due to specific changes in brain function, rather than by a placebo effect or a general effect of rTMS on brain excitability.

In this small pilot study, rTMS targets were determined by anatomical landmarks. The use of neuro-navigated rTMS based upon structural brain images may improve the effects of intervention (Ruohonen and Karhu, 2010), especially within this population as brain anatomy varies considerably across individuals (Ahdab et al., 2016). Future studies should also consider examining additional clinically-meaningful aspects of cognitive-motor performance, such as complex reaction time and/or the kinematics of gait and postural control. Finally, this study focused only on the immediate effects of rTMS intervention. As several studies have demonstrated longer-lasting effects of similar intervention within other populations (Helmich et al., 2006; Chang et al., 2010), studies with longer follow-ups are warranted.

## Author Contributions

ZL, HM, JZ, and TF designed the study. XW, ZW, YY, and LM collected the data. ZL, JZ, and VP analyzed the data and performed statistical analyses. ZL, BM, JZ, and TF drafted the manuscript. All authors contributed and approved the final version.

## Conflict of Interest Statement

The authors declare that the research was conducted in the absence of any commercial or financial relationships that could be construed as a potential conflict of interest.
